# Robot-assisted three column trans-intervertebral osteotomy by combined navigated trajectories: A feasibility study and technical report

**DOI:** 10.1016/j.bas.2025.104330

**Published:** 2025-07-17

**Authors:** Yi Huang, Jianfeng Yang, Tianhao Wang, Wenhao Hu, Xuesong Zhang, GuoQuan Zheng, Yan Wang

**Affiliations:** a Nankai University School of Medicine, Nankai University, Tianjin, 300071, China; b Department of Orthopedics, General Hospital of Chinese People's Liberation Army, Beijing, 100853, China

**Keywords:** Spinal deformity, Spinal osteotomy, Robot-assisted spine surgery, Surgical planning

## Abstract

**Introduction:**

Spinal osteotomy is indicated for malalignment and deformity, but the degree of osseous resection is mainly determined by the surgeon's experience. Navigation and robotics are techniques for the precise placement of pedicle screws.

**Research question:**

Can an innovative combined navigated trajectory (CNT) design based on a spinal robot achieve precise 3-column osteotomy.

**Materials and methods:**

The Mazor X Stealth Edition (MXSE) robotic system was used to design and execute type II trans-intervertebral osteotomy (TIO) via CNT. Preoperative CT images of a synthetic spine model and a cadaveric specimen were processed to create multitrajectory plans aligned in the sagittal plane, traversing the pedicle bases. The intraoperative workflow included a robotic setup, bone mount bridge fixation, pre- and postresection registration, and robotic trajectory drilling followed by osteotomy completion via a bone chisel.

**Results:**

After posterior element resection, the osteotomy vertebrae were successfully registered in both the synthetic and cadaveric models. Multitrajectory drilling followed by chisel combination achieved complete TIO. Quantitative analysis revealed that the deviation of the posterior vertebral wall from the preoperative plan was less than 2 mm in both specimens, with corresponding length and angle differences of −4.00 %/–1.55° (synthetic) and −6.95 %/–2.59° (cadaveric).

**Discussion and conclusion:**

Combined navigated trajectory spinal resection is a possible technique for quantitative spinal osteotomy using MXSE. Biomechanical and clinical studies are needed to further evaluate the suitability and safety of this technique.

## Highlights

• Introduced a novel **Combined Navigated Trajectories (CNT)** technique using spinal robot for quantitative spinal osteotomy enhancing precision.
• Developed a multi-trajectory strategy for controlled osteotomy closure plane, ensuring accuracy while avoiding neural structures.
• Demonstrated CNT's precision (**≤2 mm deviation**) in synthetic spine and cadaveric models, confirming real-world potential.
• Developed a structured intraoperative workflow, ensuring seamless robotic guidance and improved registration accuracy.

## 1 Introduction

Surgical treatment for adult spinal deformity to restore sagittal alignment has been associated with health-related quality of life (HRQoL)(Scheer et al., 2018; Schwab et al., 2010; Terran et al., 2013). Exquisite rationales for postoperative sagittal alignment, such as the global alignment and proportion (GAP) score, age-adjusted alignment goals, or the recent sagittal age-adjusted score (SAAS), have been reported to promote patient-reported outcomes(Lafage et al., 2016, 2022). The practical method used to restore sagittal alignment quantitatively cannot be ignored. Several techniques or combinations of techniques have been reported for restoring sagittal alignment for the newest and evidential, such as spinal osteotomy, anterior column realignment, and precontoured patient-specific rods(Ou et al., 2022; Saigal et al., 2016; Wang et al., 2023). Spinal osteotomy is a widely accepted technique for realignment and deformity correction. Quantitative osteotomy to restore sagittal alignment seems to be an attractive topic.

Several studies have suggested the safety and comparable outcomes of robot-assisted spine surgery to those of conventional techniques in recent years(Lee et al., 2022; Torii et al., 2022). Mazor X Stealth Edition (MXSE^1^) represents the 4th generation of spine robots, with anatomical trajectory guidance and real-time navigation technology(Lee et al., 2021). Its accuracy in terms of screw placement, improvement in intraoperative complications and reduction in radiation exposure have been reported. However, its cost-effectiveness is a major burden to many centers. More innovative applications might be introduced. Bederman et al. reported en bloc surgery assisted by the Mazor system(Bederman et al., 2014); thus, advanced applications beyond screw placement are pursued by spine surgeons (Massaad et al., 2021). We suggest that quantitative spinal osteotomy via combined navigated trajectories (CNTs) might be an effective utilization of MXSE.

The present study investigated the feasibility of quantitative spinal osteotomy in a synthetic spine model and cadaveric spine, utilizing an original technique named combined navigated trajectories to provide an osteotomy closure plane, which was designed via Mazor preoperative planning software and carried out via MXSE.

## 2 Materials and methods

### 2.1 Synthetic spine model and cadaveric spine

A radiopaque synthetic spine model (Sawbones foam cortical shell, SKU:1524-40) representing spinal segments L1–S1, including intervertebral discs and longitudinal ligaments, was used for initial testing. Additionally, one fresh-frozen elderly male cadaver spine sample was obtained, thawed at room temperature (25 °C), and prepared via posterior midline dissection. Ethical approval was granted by our institutional review board.

### 2.2 Robot system and trajectory design

The Mazor X Stealth Edition (MXSE; Medtronic, Dublin, Ireland) robotic system comprising a workstation, robotic arm, and intraoperative C-arm fluoroscope (GE OEC, Utah, USA) was employed. Preoperative CT scans (1-mm slice thickness) of both spine models were imported into Mazor Align software (Medtronic) for trajectory planning.

**Fig. 1:**
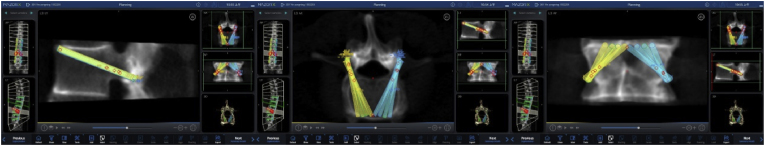
Schematic illustration of the CNT technique for spinal osteotomy in Mazor Align software. CNT, combined navigated trajectory.

Using Mazor Align software, trajectories of the CNT technique for an adjustable osteotomy plane were designed with the following principles (Fig. 1).

● The trajectories originate at the pedicle bases, forming a straight osteotomy line for chisel insertion.
● The trajectory diameter equals twice the drill-guide radius on each CT slice to maintain safe distances from nerve roots and the dural sac.
● All trajectories align sagittally, defining a uniform osteotomy closure plane
● The trajectory tips ideally extend to the anterior vertebral cortex (optional due to the 30-mm depth limitation of the standard drill guide).

After the trajectory plan was completed, it was uploaded to the MXSE workstation.

**Fig. 2:**
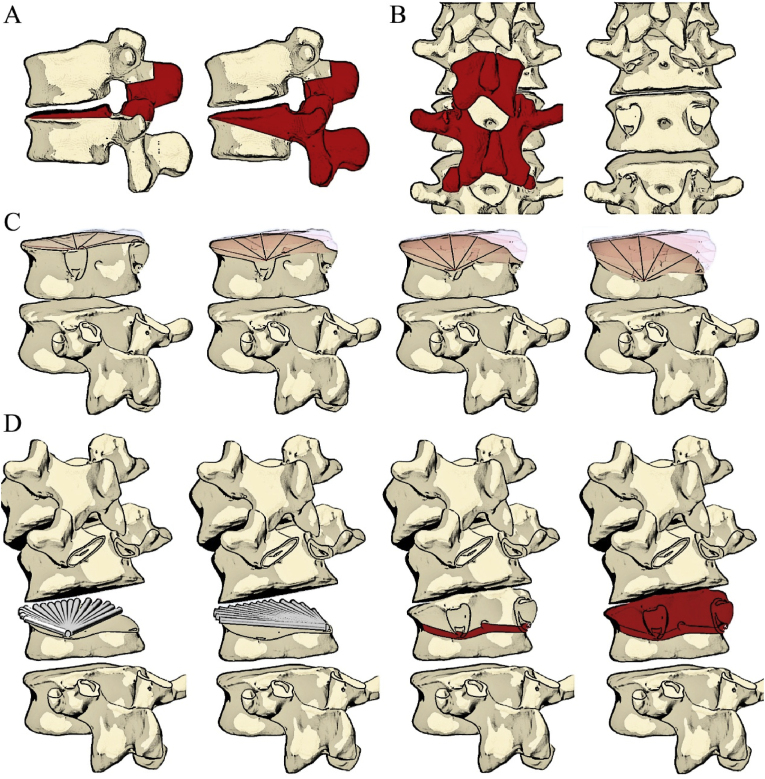
Illustrative diagram of the CNT technique in TIO. (A) Differences between Type II and Type III TIO. (B) Extent of posterior element resection needed. (C) Quantitative osteotomy precision achieved by adjusting the CNT entry points. (D) Multiple aligned trajectories consolidated into a single osteotomy plane, followed by discectomy, completing the TIO procedure. CNT, combined navigation trajectory; TIO, trans-intervertebral osteotomy.

In general, the CNT technique involves trajectory drilling via multiple robotic-guided paths, followed by consolidation with an osteotome to create a uniform osteotomy surface, achieving quantitative osteotomy, and is particularly suitable for quantitative type II to type III trans-intervertebral osteotomy (TIO) (Wang et al., 2023) procedures, which require only a single bony osteotomy surface (Fig. 2).

### 2.3 Intraoperative workflow

#### 2.3.1 Robot setup

A fiducial array ("marker grid") is mounted onto the external image intensifier of the intraoperative C-arm fluoroscope prior to surgery. The spine model or cadaveric specimen was rigidly mounted onto the robotic arm via a bone mount bridge (BMB), which included a single spinous process clamp and a dual spinous process clamp to minimize potential shifts.

#### 2.3.2 Registration progress

The registration involved three sequential steps.

Step 1 Preregistration Preparation: Initially, a collision avoidance ("3-define" or "no-fly zone") scan was performed. A sterile drape temporarily covered the reflective markers, and the robotic arm's optical camera defined a safe working area. Next, the region of interest (ROI) was marked via a navigation probe at the cranial and caudal boundaries.
Step 2 2D-3D Registration Computation: For the registration algorithm between intraoperative fluoroscopy images and preoperative CT data, another external fiducial array ("star marker") is attached to the end effector of the robotic arm, complementing the previously mounted grid on the C-arm. Optimal anteroposterior (AP) and oblique (OBL) fluoroscopic images were acquired via the C-arm encompassing all the vertebral bodies within the ROI, ensuring maximal visibility of the star marker fiducials, which were automatically transmitted to the robotic workstation. The software performs automatic spatial matching between these fluoroscopic images (including visible fiducial points) and preoperative CT data, segmentally identifying targeted vertebrae and segmentally identifying targeted vertebrae.
Step 3 Verification of Registration Accuracy: the robotic system evaluates registration accuracy on the basis of three essential criteria, "RBC principle": region (all vertebrae within the ROI are clearly captured), beats (most fiducial points visible), and clarity (optimal image resolution). Each vertebral segment in the ROI receives a registration quality rating:
● **The green spot** indicates "high-quality fluoro-CT matching" and high reliability.
● **The yellow spot** denotes "poor-quality fluoro-CT matching," which requires careful manual verification.
● **The red spot** signal registration failure, necessitating a repeat scan.

Finally, the surgeon must verify each vertebra's registration result manually on the robotic workstation to ensure reliability and accuracy. Only after confirming registration accuracy may the robotic arm be activated to execute preoperative surgical trajectories.

#### 2.3.3 Resection of posterior elements

**Fig. 3:**
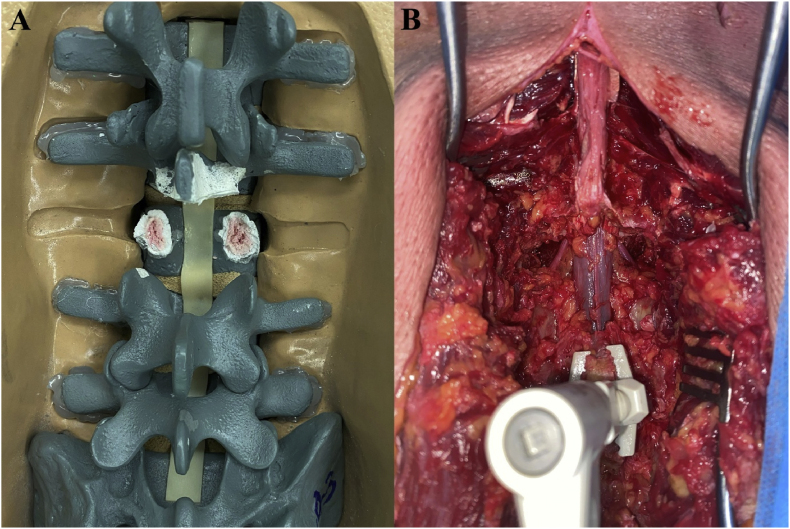
Resection of posterior elements in synthetic and cadaveric spine models. (A) Synthetic spine model fixed via dual spinous process clamps at L1 and L5; the cancellous bone of the pedicle bases is clearly visualized (pink coloured) postresection. (B) Cadaveric spine model after resection, clearly showing cancellous bone exposure at the pedicle bases.

For the synthetic spine model, L3 was determined for TIO, and a dual spinous process clamp was used at L1 and L5 for mounting. The L3 spinous process, bilateral lamina, pedicles and L2 partial spinous process, lamina, and inferior process were subsequently resected, exposing the cancellous bone of the pedicle bases (Fig. 3A).

For the cadaveric spine, L4 was used for TIO, and a single spinous process clamp was used at L5 for mounting. The L4 vertebra was exposed via dissection lateral to the transverse process. Posterior elements, including the spinous process, bilateral lamina, and adjacent facet joints, were removed, and the L3 partial spinous process, lamina, and inferior process were also resected for routine predecompression. The L4 pedicles were subsequently removed, and the spinal canal was opened laterally, exposing the cancellous bone of the pedicle bases (Fig. 3B). After the resection of posterior elements, a second registration process was conducted for accuracy due to anatomical modifications (TIO level and TIO +1 level) and possible displacement.

### 2.4 Accuracy grading and analysis for osteotomy resection

On the basis of the pedicle screw position grading by Ravi (Ravi et al., 2011) and considering the repositioning accuracy of the 4th generation of spine robot arms, we modified the grading system for osteotomy resection with CNT: I, the posterior wall of the resected vertebra was equal to the preoperative plane; II, the posterior wall of the resected vertebra differed by less than 2 mm according to the plan; III, the posterior wall of the resected vertebra differed by 2–4 mm; IV, the posterior wall of the resected vertebra differed by more than 4 mm. The osteotomy closure length difference ratio is defined as the percentage difference between the actual midline length of the posterior vertebral wall after osteotomy (*Len*_*osteo*_) and the planned midline length of the posterior vertebral wall after osteotomy (*Len*_*plan*_), both normalized by the total midline length of the posterior vertebral wall (*Len*_*total*_), as shown below:

$$\text{Closure}\;\text{length}\;\text{difference}\;\text{ratio}=\left(Len_{osteo}/Len_{total}\;–\;Len_{plan}/Len_{total}\right)\times 100\%$$

The difference in the closure angle was calculated as the difference between the actual osteotomy angle and the planned osteotomy angle. The planned angle is determined by the arctangent length of the planned posterior wall midline divided by the anterior‒posterior diameter of the superior endplate (*Len*_*endplate*_). Similarly, the actual angle is determined from the actual posterior wall length. The difference between the two angles yields the closure angle difference, as shown below:

$$\text{Closure}\;\text{Angle}\;\text{Difference}=\arctan\left(Len_{osteo}/Len_{endplate}\right)–\arctan\left(Len_{plan}/Len_{endplate}\right)$$

## 3 Results

### 3.1 Registration before and after resection of posterior elements

**Fig. 4:**
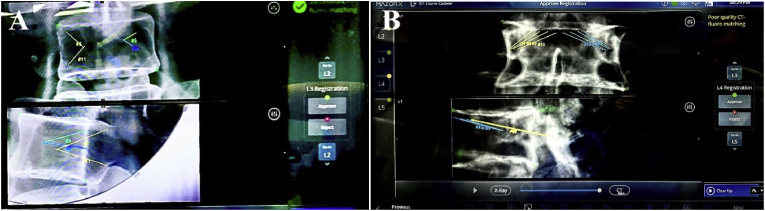
Registration results of the MXSE robotic system following posterior resection. (A) Synthetic spine model: Osteotomy vertebra without posterior elements and pedicles successfully recognized (green spot, high-quality registration). (B) Cadaveric spine model: Osteotomy vertebra recognized with caution (yellow spot, moderate-quality registration) after adjustment of fiducial markers. MXSE, Mazor X Stealth Edition.

For the synthetic spine model, registration of the ROI, including L1--L5, before resection was performed successfully with all the green spots, which illustrated that the Sawbones foam cortical shell synthetic spine model can be recognized by the MXSE system. The second registration after resection of the posterior elements, the L2 vertebra without a partial spinous process, lamina, and inferior process was successfully recognized with the green spot; the L3 vertebra without a spinous process, bilateral lamina and pedicles was also successfully recognized with the green spot, and after comparison, it was approved for further operation (Fig. 4A).

For the cadaveric spine, registration of the ROI, including L2--L5, before resection was performed successfully with all the green spots. The second registration after resection of the posterior elements, L2, L3 and L5, was successfully recognized with the green spot; the L4 vertebra without the spinous process, bilateral lamina and pedicles initially failed to be recognized with the red spot. After adjusting the 3D marker to reduce its occlusion to the L4 cortex, the L4 vertebral body was recognized with a yellow spot (Fig. 4B). After the comparison, it was also approved for further operation. The registration accuracy results after posterior element resection are summarized in Table 1.

**Table 1:** Results of the second registration after resection of posterior elements. ROI: region of interest; TIO: trans-intervertebral osteotomy; BMB: bone mount bridge; CT: computerized tomography.

Object	ROI	TIO level	BMB level	BMB distance to TIO/BMB number	Registration quality of anatomical modification level		Ratio of high-quality fluoro-CT matching
					TIO+1 level	TIO level	
Synthetic spine model	L1-L5	L3	L1, L5	2/2	green spot	green spot	100 %
Cadaveric spine	L2-L5	L4	L5	1/1	green spot	yellow spot	75 %

### 3.2 Trajectory drilling, combinations, and confirmation

**Fig. 5:**
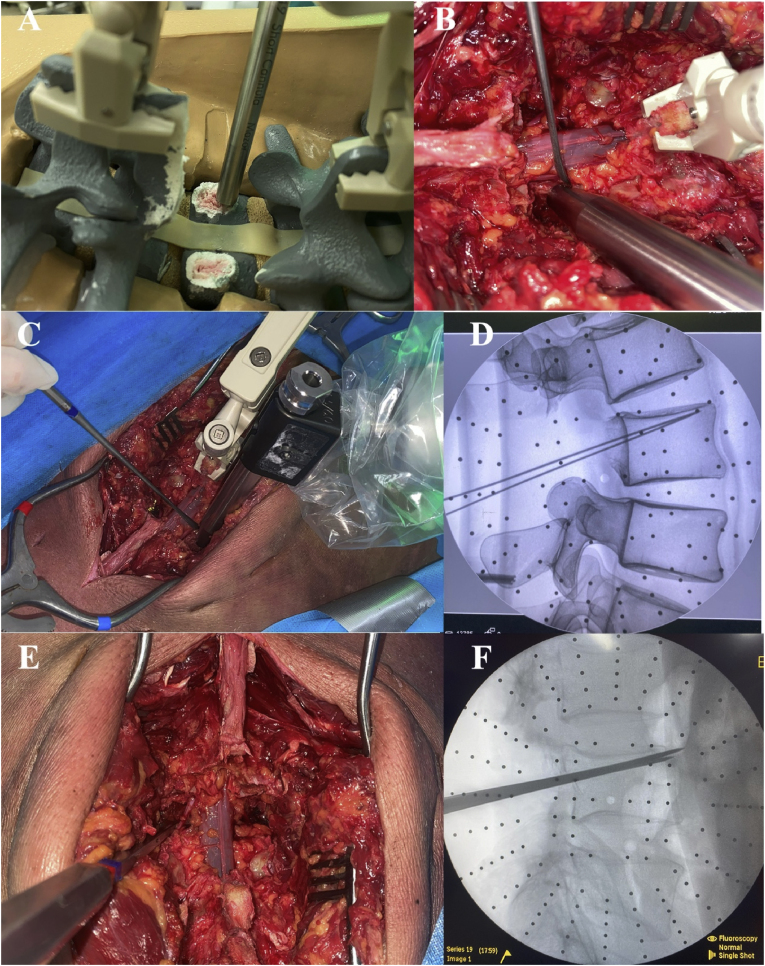
Robotic-guided trajectory drilling and osteotomy consolidation procedure. (A) Drill guide accurately positioned in cancellous bone at the pedicle bases of L3 in the synthetic spine model. (B) Robotic drill guide placement in the cadaveric spine, demonstrating intraoperative nerve root protection via a surgical hook. (C) Confirmatory image showing successful drill guide navigation in the cadaveric spine. (D) Verification of trajectory placement via intraoperative C-arm fluoroscopy with K-wire insertion (synthetic model). (E) Bone chisel consolidation of multiple trajectories to complete the osteotomy surface. (F) Bilateral insertion of the bone chisel confirmed via fluoroscopy, verifying successful TIO completion. TIO, trans-intervertebral osteotomy.

The surgeon activates the surgical arm by selecting the planned trajectory at the desired level to be executed. Once the robotic arm was in position, the surgeon inserted the working cannula of the appropriate length. The robotic arm positioned the working cannula precisely over planned trajectories. Soft tissue was retracted gently to avoid pressure on system. A drill guide with antiskive teeth was used to gently impact the cancellous bone at the pedicle base, which was verified manually by applying gentle torque for stability(Avrumova et al., 2021). The planned trajectory successfully guided the drill guide with the teeth inserted in the cancellous bone of the L3 pedicle bases in the synthetic spine model (Fig. 5A). For the cadaveric spine, the planned trajectory successfully guided the drill guide, and we simulated the intraoperative protection of the nerve root with a hook (Fig. 5B and C). Using a 3-mm high-speed drill (Powerease, Medtronic), multiple pilot trajectories were created under robotic guidance. Fluoroscopy confirmed the trajectory positions via K-wire insertion (Fig. 5D). Throughout robotic trajectory execution, no instances of instrument skiving or unintended robotic arm displacement occurred. Real-time visual confirmation consistently indicated stable positioning and adherence to the preoperative trajectory plans. After all trajectories were completed, the robotic equipment was removed from the surgical field.

### 3.3 Trans-intervertebral osteotomy via combined navigated trajectories

The bone chisel was used to combine the drilled trajectories (Fig. 5E) and then to osteoclasts to the anterior cortex and lateral walls of the vertebral body. The osteotomized part of the vertebra was subsequently removed. With the insertion of the bone chisel bilaterally, C-arm fluoroscopy was used to confirm TIO of the cadaveric spine, as shown in Fig. 5F.

The quantitative accuracy of osteotomy resection was evaluated against that of preoperative planning (Table 2). In the synthetic spine model, the calliper measurement indicated a posterior wall deviation of 1.2 mm from the preoperative plane, classified as grade II accuracy, resulting in a closure length difference ratio of −4.00 % and a closure angle difference of −1.55°.

**Table 2:** Quantitative assessment of posterior wall resection accuracy. TIO, trans-intervertebral osteotomy.

Object	TIO level	Sagittal endplate length (mm)	Total posterior length (mm)	Planned resection length (mm)	Actual resection length (mm)	Length difference (mm)	Closure length difference ratio	Closure angle difference (°)	Modified Ravi grading
synthetic spine model	L3	39.7	30.0	14.1	12.9	1.2	−4.00 %	−1.55	II
cadaveric spine	L4	33.6	25.9	15.4	13.6	1.8	−6.95 %	−2.59	II

**Fig. 6:**
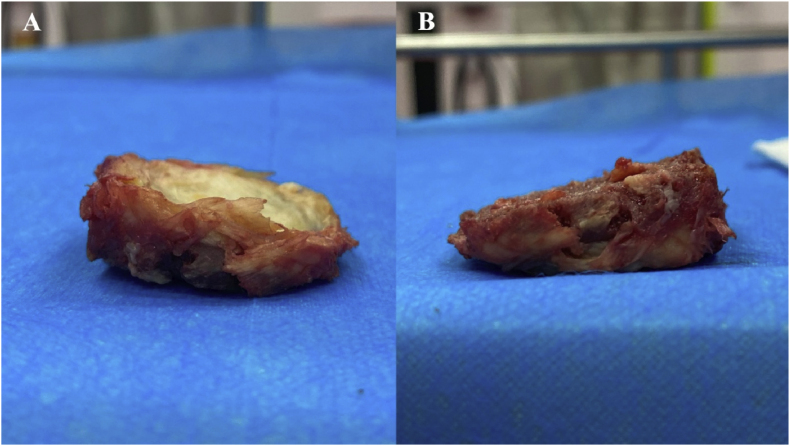
The resected vertebral segment in the cadaveric spine was completely removed after robotic-guided osteotomy.

The resected part of the cadaveric vertebral body was removed entirely after discectomy and detachment (Fig. 6). After measurement by a calliper, the posterior wall of the resected vertebra differed from the plan by 1.8 mm, which is a Grade II quantitative resection by our grading system, corresponding to a closure length difference ratio of −6.95 % and an angle difference of −2.59°.

## 4 Discussion

For various types of spine surgery—tumors, trauma, degeneration, and deformities—the use of a pedicle screw (PS) system remains the most commonly applied technique. The initial development of spinal robots was largely focused on assisting PS insertion, reflecting its widespread use in clinical practice(Alluri et al., 2021). The true value of spinal robotic systems lies in their stereotactic capability, yet their use has been mostly limited to pedicle screw insertion—a common but not necessarily cost-effective application with freehand screw placement remains widely accepted(Torii et al., 2022). Current platforms remain task driven and standardized. Future directions should expand their utility toward more complex, high-impact procedures beyond instrumentation. Innovative and more complex utilization of the MXSE system has been investigated and reported(Good et al., 2022; Pham et al., 2022). Recently, Satin et al. described the planning function of robot-assisted osteotomy simulation in revision surgeries but did not demonstrate the actual implementation of osteotomy execution(Satin et al., 2022). The present study explores robot-assisted spinal osteotomy, aiming to address the technical and quantitative aspects of sagittal alignment restoration, given its established impact on improving outcomes(Schwab et al., 2010; Bridwell et al., 2003).

Given the potential benefits of precise osteotomy in restoring sagittal alignment and optimizing bony contact to reduce the risk of pseudarthrosis, such efforts can be traced back to the early adoption of image-guided navigation techniques, first reported in a case study by Metz et al., where navigated drilling was utilized for marking and refining osteotomy borders (Metz et al., 2008). Faundez et al. described a navigated osteotomy technique using the O-arm navigation system to optimize bone resection angles for precise closure to minimize the risk of pseudarthrosis(Faundez et al., 2015). Compared with image-guided navigation osteotomy alone, our CNT approach using a fourth-generation spinal robotic system has two principal advantages: reducing instrument deviation, the robotic arm's end-effector drill guide provides stable physical guidance, substantially reducing deviations caused by surgeon hand tremors compared with conventional navigation-guided freehand osteotomies, and enhancing surgical safety. In conventional navigation-based osteotomies, surgeons must frequently shift their gaze away from the surgical field to the navigation screen, increasing risks, especially around critical neural structures. Conversely, the MXSE system allows surgeons to maintain direct visualization of the surgical site, using robotic trajectory guides as a primary safety measure and navigation as a redundant safety verification.

The registration process is crucial for the accurate navigation outcomes of a spinal robot(O'Connor et al., 2021). Intersegmental micromotion presents a unique challenge for maintaining registration fidelity across multiple vertebral levels, whereas late-generation spinal robots integrate enhanced stereotactic algorithms and rigid mechanical tracking systems, such as the BMB, as a fixed hard registration reference for mechanical real-time tracking of vertebrae segmentally, in contrast to the optical dynamic tracking used in knee arthroplasty systems for the femoral and tibial segments. Importantly, these rigid tracking systems have constraints: the number of BMB fixation points and the distance between the BMB and the surgical target significantly influence registration stability and execution accuracy, whereas no prior studies have quantitatively reported the relationship between robot mounting device positioning and navigational reliability. Vibrations from traditional decompression techniques, such as posterior resection with rongeurs or osteotomes, can significantly affect robotic registration accuracy. In this study, the BMB was removed during preosteotomy decompression, and subsequent reinstallation and reregistration were essential. Our protocol involved two-step registration: the initial step ensured anatomical recognition, and the second step confirmed accuracy after structural changes. Registration failure was associated primarily with landmark loss, displacement, and fiducial occlusion. Maintaining BMB fixation without reregistration after decompression may reduce accuracy. Future studies should explore how such effects are related to the BMB configuration, including the fixation number and proximity to the surgical target. Since dual registration increases the operative time, performing decompression with low-vibration tools such as ultrasonic bone scalpels—while keeping the BMB fixed—may offer a more efficient solution without compromising precision.

The possibility for quantitative osteotomy, or more radical, quantitative correction, on the basis of the hypotheses of accurate prediction of postoperative alignment(Koller et al., 2018) and the ratio of closure (Diebo et al., 2016), seems challenging because of the mobility of intervertebral segment motion, self-compensation mechanism and other factors (Horn et al., 2018). Our study deliberately set aside the complexities of postoperative closure and instead focused on how to quantify and evaluate the osteotomy itself. We propose an adapted grading system for osteotomy accuracy based on the Ravi classification and introduce novel quantitative metrics, including the closure length difference ratio and closure angle difference. These parameters offer a refined framework for assessing osteotomy precision in the era of robotic-assisted spine surgery. Schwab (Schwab et al., 2014) and Wang (Wang et al., 2023) proposed osteotomy classification systems based on the vertebral body and intervertebral space, respectively, both highlighting the modular nature of spinal osteotomy, where the degree of correction is constrained by the amount and geometry of osseous resection. Quantitative TIO, involving only a single bony resection surface—the caudal endplate of the cranial vertebra—offers a theoretical precision advantage over PSO, whose two-plane configuration inherently doubles the margin of error. Quantitative osteotomy can be performed by switching the osteotomy plane from the caudle of the pedicle to the cranial part of the upper endplate (Fig. 2C). Our results revealed an osteotomy resection in which the posterior wall of the resected vertebra differed from the plan by less than 2 mm, corresponding to Grade II accuracy under the modified Ravi classification (Ravi et al., 2011; Gelalis et al., 2012). This level of precision was achieved despite anatomical alterations and reregistration challenges, suggesting that the CNT trajectory design—guided by the pedicle base and aligned in a single sagittal plane—can offer reliable intraoperative control over the osteotomy geometry. Complementary metrics—including the closure length difference ratio (−4.00 % and −6.95 %) and closure angle difference (−1.55° and −2.59°)—further support the reliability of CNT-based resection. These quantifiable indicators provide a practical framework for evaluating osteotomy precision and set the stage for future refinements in drill diameter, trajectory design, and angular control. This study reflects the emerging paradigm in spinal robotics, where trajectory design may become a cornerstone of future technologies in deformity correction. Although robotic-guided saws or drills are effectively employed in TKA, their direct application in spinal osteotomy poses significant safety concerns because of vibration-induced tissue injury, trajectory deviation, and the risk of spinal cord injury. Thus, we intentionally avoided direct robotic saw/drill use for osteotomy, instead opting for precisely navigated trajectories combined manually via a bone chisel.

The limitations of the present study are the pilot study nature, which represents an initial feasibility study limited by the number of samples due to practical constraints. Despite achieving osteotomy deviations of less than 2 mm in both models, further refinement is necessary to establish true quantitative precision by analysing the impact of parameters such as drill diameter, trajectory number, and osteotomy plane planning on surgical accuracy, especially considering that the CNT technique is not inherently minimally invasive and requires posterior resection, with postosteotomy factors—including bone compression, closure dynamics, and instrumentation rod angle—potentially influencing alignment outcomes and thus warrants future biomechanical investigation. While two-step registration was employed to ensure navigational accuracy before and after anatomical modification, limitations remain in the anatomical recognition capability of the MXSE system—particularly after posterior element removal—due to its sensitivity to landmark loss, structural displacement, and fiducial marker occlusion, all of which may compromise intraoperative registration reliability and therefore need further investigation.

## 5 Conclusion

CNT spinal resection is a possible technique for quantitative spinal osteotomy via MXSE and may serve as a foundation for future robot-assisted correction of complex spinal deformities. Biomechanical and clinical studies are needed to further evaluate the suitability and safety of this technique.

## Ethics approval and consent to participate

This study was approved by the Ethics Committee of the Chinese PLA General Hospital, and all participants provided written informed consent. All procedures were performed in accordance with relevant guidelines.

## Availability of data and materials

The datasets used or analysed during the current study are available from the corresponding author upon reasonable request.

## Funding

This work was supported by grants from the Beijing Municipal Natural Science Foundation (Grant No. 7212093).

## Declaration of competing interest

The authors declare that they have no known competing financial interests or personal relationships that could have appeared to influence the work reported in this paper.

## Glossary

AP: anteroposterior
BMI: body mass index
BMB: bone mount bridge
CNT: combined navigated trajectories
CT: computerized tomography
MXSE: Mazor X Stealth Edition
OBL: oblique
PS: pedicle screw
PSO: pedicle subtraction osteotomy
ROI: region of interest
TIO: trans-intervertebral osteotomy
